# Providing antiretroviral therapy to all who are HIV positive: the clinical, public health and programmatic benefits of *Treat All*

**DOI:** 10.1002/jia2.25078

**Published:** 2018-02-13

**Authors:** Nathan Ford, Marco Vitoria, Meg Doherty

**Affiliations:** ^1^ HIV/AIDS Department and Global Hepatitis Programme World Health Organization Geneva Switzerland

**Keywords:** antiretroviral therapy, guidelines, public health approach, Treat All, when to start, World Health Organization

In September 2015 the World Health Organization (WHO) recommended antiretroviral therapy (ART) be offered to all adults living with HIV, regardless of WHO clinical stage and at any CD4 cell count 1. This recommendation was supported by more than two decades of research into the clinical and public health benefits of immediate ART 2.

WHO had previously assessed the benefits of *Treat All* in mid‐2013 by reviewing the results of observational studies and mathematical models that suggested starting ART as soon as possible would reduce HIV‐related morbidity, mortality and incidence. After deliberations lasting almost 2 days, the 2013 Guideline Development Group concluded that there were insufficient grounds to recommend a global policy of *Treat All*, and instead recommend that the threshold for stating ART be raised from d350 cells/mm^3^ to d500 cells/mm^3^ 3. However, immediate, lifelong ART was recommended for HIV serodiscordant couples and HIV‐positive pregnant women to prevent onward transmission 4 and, importantly in the case of the latter, to simplify service delivery as pioneered in Malawi 5. Immediate ART was also recommended for all children under 5 years, again based on programmatic ease and efficiencies and to reduce early death by starting ART early among children, half of whom will die in the first 2 years following birth without treatment 6. Finally, it was recommended to start ART in all individuals with TB or hepatitis B coinfections, given the established clinical benefits. Taken together, these recommendations meant that the majority of people living with HIV in high HIV‐burden settings were already eligible for ART.

The formulation of guideline recommendations at WHO follows a careful assessment of all available evidence across a number of domains. Clinical evidence of benefits and harms, appraised through systematic reviews and the GRADE framework, forms the basis for deliberation, but also consider values and preferences, feasibility, acceptability, resources and equity 7. When the next WHO 2016 Guideline Development Group was convened in June 2016, the START and TEMPRANO trials had both recently concluded and investigators from both trials made the main findings available to the Guideline Development Group ahead of publication 8, 9. In addition to the evidence of clinical benefit from these two randomized trials and 17 observational studies, a number of additional inputs were provided to support the decision to recommend *Treat All*, including: country experience in providing immediate ART for HIV serodiscordant couples, key populations, and pregnant women; cost effectiveness modeling, surveys of community values and preferences, and feasibility studies 10. In considering the potential benefits of *Treat All*, particular value was placed on the potential reduce the substantial loss to care among _pre‐ART_ patients who had been diagnosed with HIV infection but were not yet eligible to start ART. A systematic review of retention in care prior to starting ART found that 41% of patients were lost to care in the step between testing HIV positive and being assessed for eligibility for treatment (by CD4 cell count or clinical stage), and 32% of patients were lost between determination of eligibility and starting ART 11 Rapid initiation of ART, including starting ART on the same day that a positive HIV diagnosis is made, is now strongly by WHO as a way to reduce losses to care during this pre‐ART period 12, 13.

As of the end of 2017, 70% of low and middle‐income countries had adopted the *Treat All* policy, demonstrating a high level of acceptability of this recommendation by policy makers (Figure 1) 14. Despite fears expressed previously with respect to expanding ART eligibility, no major stock‐outs of ART or other essential supplies have been reported to be associated with the implementation of *Treat All*. Clinical and implementation research continue to provide important insights into the effectiveness of treating all persons living with HIV. Data from the START trial have found that immediate ART led to improved quality of life 15, and the programmatic benefits of early and rapid ART initiation in terms of reducing pre‐ART loss‐to‐care can be substantial 13, 16, 17.

**Figure 1:**
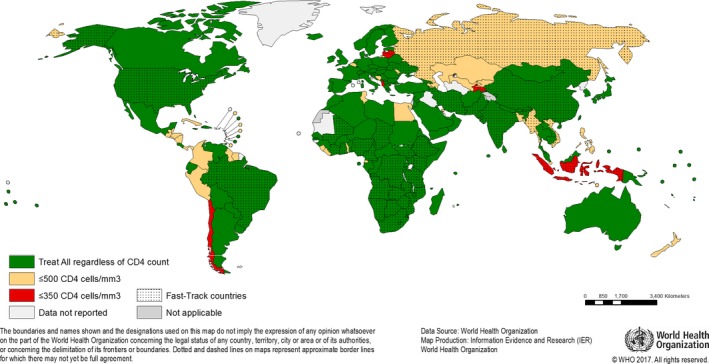
Uptake of WHO policy for Treat All ART initiation among adults and adolescents living with HIV (situation as of November 2017).

Notwithstanding these benefits, there are important programmatic challenges to achieving the full benefits of *Treat All*. The first randomized trial to assess the effect of immediate ART at the population level was unable to demonstrate a reduction in HIV incidence, a finding explained by the challenges faced in ensuring adequate linkage to care 18. This finding is echoed by cohort data demonstrating that despite a progressive guideline evolution toward earlier initiation of ART in recent years, still approximately a third of people starting ART do so at a CD4 cell count <200 cells/mm^3^ 19.

Notwithstanding, WHO and country guidelines recommending *Treat All* are an important first step, but the clinical, public health and programmatic benefits will only be realized if progress is made in testing people earlier, ensuring effective linkage, and maximizing ARV adherence and retention in HIV care over the long term. These are the key challenges ahead in making progress towards the goal of achieving epidemic control in the next decade.

## Authors′ contribution

NF Wrote the first draft of this Viewpoint. All authors contributed equally to the writing of this manuscript and approved the final version.

## Competing Interest

None.

## References

[1] World Health Organization . Guideline on when to start antiretroviral therapy and on pre‐exposure prophylaxis for HIV. World Health Organization Geneva: WHO; 2015. 26598776

[2] Eholie SP , Badje A , Kouame GM , et al. Antiretroviral treatment regardless of CD4 count: the universal answer to a contextual question. AIDS Res Ther. 2016;13:27. 27462361 10.1186/s12981-016-0111-1 PMC4960900

[3] World Health Organization . Consolidated guidelines on the use of antiretroviral drugs for treating and preventing HIV infection: recommendations for a public health approach. Geneva: WHO; 2013. 24716260

[4] Cohen MS , Chen YQ , McCauley M , et al. Prevention of HIV‐1 infection with early antiretroviral therapy. N Engl J Med. 2011;365(6):493–505. 21767103 10.1056/NEJMoa1105243 PMC3200068

[5] Schouten EJ , Jahn A , Midiani D , et al. Prevention of mother‐to‐child transmission of HIV and the health‐related millennium development goals: time for a public health approach. Lancet. 2011;378(9787):282–4. 21763940 10.1016/S0140-6736(10)62303-3

[6] Newell ML , Coovadia H , Cortina‐Borja M , et al. Mortality of infected and uninfected infants born to HIV‐infected mothers in Africa: a pooled analysis. Lancet. 2004;364(9441):1236–43. 15464184 10.1016/S0140-6736(04)17140-7

[7] World Health Organization . WHO Handbook for Guideline Development. 2nd Ed. Geneva: WHO; 2014.

[8] Group ISS , Lundgren JD , Babiker AG , et al. Initiation of Antiretroviral Therapy in Early Asymptomatic HIV Infection. N Engl J Med 2015; 373(9): 795–807. 26192873 10.1056/NEJMoa1506816 PMC4569751

[9] Group TAS , Danel C , Moh R , et al. A Trial of Early Antiretrovirals and Isoniazid Preventive Therapy in Africa. N Engl J Med 2015; 373(9): 808–22. 26193126 10.1056/NEJMoa1507198

[10] World Health Organization . Global report on early warning indicators of HIV drug resistance. Geneva: WHO; 2016.

[11] Rosen S , Fox MP . Retention in HIV care between testing and treatment in sub‐Saharan Africa: a systematic review. PLoS Med. 2011;8(7):e1001056. 21811403 10.1371/journal.pmed.1001056 PMC3139665

[12] World Health Organization . Guidelines for managing advanced HIV disease and rapid initiation of antiretroviral therapy. Geneva: WHO; 2017 Available http://www.who.int/hiv/pub/toolkits/advanced-HIV-disease-policy/en/ 29341560

[13] Ford N , Migone C , Calmy A , et al. Benefits and risks of rapid initiation of antiretroviral therapy. AIDS. 2018;32(1):17–23. 29112073 10.1097/QAD.0000000000001671 PMC5732637

[14] World Health Organization . Treat All: policy adoption and implementation status in countries. Geneva: November 2017. Available http://apps.who.int/iris/bitstream/10665/259532/1/WHO-HIV-2017.58-eng.pdf.

[15] Lifson AR , Grund B , Gardner EM , et al. Improved quality of life with immediate versus deferred initiation of antiretroviral therapy in early asymptomatic HIV infection. AIDS. 2017;31(7):953–63. 28121710 10.1097/QAD.0000000000001417 PMC5373969

[16] Bor J , Fox MP , Rosen S , et al. Treatment eligibility and retention in clinical HIV care: A regression discontinuity study in South Africa. PLoS Med. 2017;14(11):e1002463. 29182641 10.1371/journal.pmed.1002463 PMC5705070

[17] Tymejczyk O , Brazier E , Yiannoutsos C , Wools‐Kaloustian K , Althoff K , Crabtree‐Ramirez B , et al. The impact of HIV treatment guideline expansion on the timeliness of ART initiation following enrollment in HIV care. 21st International Workshop on HIV and Hepatitis Observational Databases, Lisbon 2017. Abstract 30..

[18] Iwuji CC , Orne‐Gliemann J , Larmarange J , et al. Universal test and treat and the HIV epidemic in rural South Africa: a phase 4, open‐label, community cluster randomised trial. Lancet HIV. 2017; [Epub ahead of print]. https://doi.org/10.1016/S2352-3018(17)30205-9. 10.1016/S2352-3018(17)30205-9 29199100

[19] Anderegg N KOoboI‐GAaC . Immunodeficiency at the start of combination antiretroviral therapy in low‐, middle‐ and high‐income countries. 21st International Workshop on HIV and Hepatitis Observational Databases, Lisbon, Portugal, 30 March–1 April 2017. Abstract 12.

